# A SAR-Based Classification Model for Assessing Pesticide Toxicity to *Apis mellifera*

**DOI:** 10.3390/jox16040130

**Published:** 2026-07-11

**Authors:** Nadia Iovine, Anna Lombardo, Alessandra Roncaglioni, Emilio Benfenati

**Affiliations:** Department of Environmental Health Science, Istituto di Ricerche Farmacologiche Mario Negri IRCCS, Via Mario Negri 2, 20156 Milano, Italy; anna.lombardo@marionegri.it (A.L.); alessandra.roncaglioni@marionegri.it (A.R.); emilio.benfenati@marionegri.it (E.B.)

**Keywords:** classification model, honey bees, LD_50_, SAR, SARpy, environmental toxicology

## Abstract

Pollinators are essential for maintaining ecosystem stability and agricultural productivity, yet their populations are in decline due to various stressors, including parasites, pathogens, climate change and pesticide exposure. Protecting pollinators has become a priority for environmental safety and food security. Regulatory authorities, including the European Food Safety Authority (EFSA), the Environmental Protection Agency (EPA) and the Organisation for Economic Co-operation and Development (OECD), have guidelines for pesticide risk assessment, but conventional testing methods are costly and time-consuming, limiting their applicability to large chemical datasets. Computational approaches, such as Structure–Activity Relationship (SAR) models, offer efficient alternatives by enabling the rapid screening of pesticides for potential toxicity to pollinators. In this study, we used a dataset of 357 compounds to develop a classification model based on structural alerts to predict oral acute toxicity in *Apis mellifera*. The model showed a higher Matthews Correlation Coefficient in the training set (0.82), with a moderate decay in the test set (0.56) likely due to applicability domain limits. Despite this, high balanced accuracy (0.80) and sensitivity (0.79) in the test set confirm the model as a reliable tool for the toxicological screening of pesticides.

## 1. Introduction

Honey bees (*Apis mellifera*) are among the most important pollinators in both natural ecosystems and agricultural landscapes [1,2]. Their role in the pollination of a wide variety of crops and wild plants is essential for biodiversity conservation and global food production. It is estimated that around 75% of the world’s food crops depend, at least in part, on pollinators, with honey bees contributing significantly to the reproduction and yield of fruits, vegetables, nuts and oilseeds [3,4]. The decline of bee populations observed in recent decades has therefore raised serious concerns worldwide, as it threatens not only ecosystem stability but also food security and agricultural productivity [2,5,6,7].

Among the multiple stressors linked to honey bee decline, including habitat fragmentation, pathogens, parasites and climate change, the use of pesticides is recognized as one of the most critical anthropogenic factors [7,8,9]. Pesticides are widely used in modern agriculture to control weeds, insects, and fungal pathogens, and they are typically classified into four main categories: insecticides, herbicides, fungicides and acaricides. Pesticides can affect honey bees through multiple mechanisms, impacting both colonial species, such as honey bees (*A. mellifera*) and solitary bees. Acute exposure may directly reduce survival rates, leading to colony losses or individual mortality. However, even at sub-lethal levels, pesticides can have profound effects on honey bee health and functioning [10,11]. Pesticides can disrupt the nervous system, alter honey bee behavior more broadly, affecting communication, navigation, and social interactions within colonies, which in turn reduces the efficiency of pollination and resource collection [12,13]. Additionally, exposure can compromise the immune system, making honey bees more susceptible to pathogens and parasites [13,14]. Emerging evidence suggests that pesticides may also disturb the gut microbiota, which plays a crucial role in nutrition, immunity, and overall health [15,16]. The combined impact of these effects can weaken colonies over time and threaten pollinator populations at a landscape scale.

To mitigate these risks, regulatory authorities such as the European Food Safety Authority (EFSA) and the U.S. Environmental Protection Agency (EPA) have developed specific guidelines for the environmental risk assessment of pesticides [17,18] and the European Commission established a specific Action Plan to protect pollinators [19]. The Organisation for Economic Co-operation and Development (OECD) published specific guidelines that include standardized in vivo toxicity tests, such as the OECD Test Guidelines 213 [20] and 214 [21], which evaluate acute oral and contact toxicity in adult bees. While these tests are essential for characterizing the hazards associated with chemical exposure, they are also resource-intensive, time-consuming and ethically problematic due to the use of live animals [22,23,24].

In this context, New Approach Methodologies (NAMs) are gaining momentum as alternative or complementary strategies for chemical safety assessment [25,26]. Among them, in silico models, such as SAR (Structure–Activity Relationship) and Quantitative Structure–Activity Relationship (QSAR) models, offer a promising way to predict the toxicity of chemical compounds based on their molecular structure and physicochemical properties [22,24,27]. (Q)SAR models can be used to estimate toxicological endpoints without the need for experimental testing, thus reducing animal use and enabling the rapid screening of large chemical inventories. These computational tools align with the 3Rs principles (Replacement, Reduction and Refinement) and support regulatory initiatives aimed at increasing efficiency and sustainability in risk assessment processes [28].

The use of in silico models for predicting pesticide toxicity to bees is still relatively limited compared to other species, but recent efforts have demonstrated their potential. Multiple studies have demonstrated that in silico methods can effectively predict and classify pesticides as toxic or non-toxic to bees, achieving acceptable levels of accuracy, sensitivity and specificity [29,30,31,32,33,34]. More recently, new approaches and algorithms have been introduced, such as Graph Neural Networks, Deep Learning, and Graph kernels, using multiple combined sources of data. This allowed the introduction of novel methods to deal with the chemical structures, referring to graphs, for instance, but also exploring the use of degradability values as a support to the assessment [29,35,36]. Moreover, curated databases [37,38], datasets and tools [39,40,41,42] are increasingly available, allowing researchers to use and/or build reproducible and interpretable models. Such models can serve as decision-support tools in early stages of pesticide development or regulatory evaluation, helping to identify hazardous compounds before they are tested or released into the environment.

In this study, we describe the development of a binary classification model designed to predict the toxicity of pesticides to honey bees (*A. mellifera*). The model is based on hundreds of pesticides, covering insecticides, herbicides, fungicides and acaricides. By integrating curated toxicological data with Structural Alerts (SAs), our goal is to provide a reliable in silico model that can support risk assessors and researchers in identifying potentially harmful pesticides. This work contributes to the growing body of evidence supporting the use of computational models in ecotoxicology and highlights their relevance in protecting pollinators and promoting sustainable agricultural practices.

## 2. Materials and Methods

### 2.1. Data Collection

Data were collected from two publicly available databases (OpenFoodTox and ECOTOX) and one commercial database (Pesticide Properties Database, PPDB), following the criteria described in the OECD TG 213 [20].

OpenFoodTox [37]: Version 6 of the database was obtained from the Zenodo repository (https://zenodo.org/records/8120114, accessed on 14 March 2025), and specific filters were applied to extract relevant records, as detailed in Table 1. It is important to highlight that the data available for *A. mellifera* in this database refer exclusively to adults, as this was one of the criteria for data selection.

ECOTOX [38]: Data were collected from the online database (https://cfpub.epa.gov/ecotox/search.cfm, accessed on 14 March 2025) by selecting *A. mellifera* as the species and LD_50_ as the endpoint. A series of filters (Table 2) was applied to refine the dataset and ensure the relevance and reliability of the collected data.

PPDB [43]: Data on the 48 h oral LD_50_ for *A. mellifera* were collected from this database (https://sitem.herts.ac.uk/aeru/ppdb/, accessed on 19 September 2024), which provides information on data origin and reliability, even if lacking the level of detail found in the previously used source. For this study, only entries classified as “Verified data” and “Verified data used for regulatory purposes” were included in the analysis.

Table 3 contains the number of records collected and the number of data records obtained after the application of filters.

### 2.2. Data Analysis

The data on toxicity towards honey bees collected from the three databases were analyzed to search for chemical information, such as SMILES and CAS numbers, useful for the final dataset. For some of the collected compounds, CAS numbers and SMILES representations were initially unavailable. The missing information was retrieved and subsequently standardized using an in-house tool (which will be made available within VEGAHUB—www.vegahub.eu), the CHEMICAL SMILER, which also performed structure neutralization.

Data from the three databases were merged, and a data cleaning procedure was subsequently applied. First, each record was classified according to toxicity based on a threshold of 100 µg/bee, in line with established guidelines [17,20]:

- Toxic: LD_50_ < 100 µg/bee
- Non-toxic: LD_50_ ≥ 100 µg/bee

Next, duplicate entries were removed using a conservative approach adopted due to the limited availability of data:

- When multiple identical LD_50_ values were reported for the same substance, only a single record was retained.
- In cases where duplicate entries reported different LD_50_ values and the ratio between the highest and lowest values was less than 5, we applied the following rules. The factor of 5 was chosen based on a survey regarding the accuracy and reproducibility of the experimental values for pesticides [44]. If all LD_50_ values were reported with the ‘=’ qualifier, the lowest value was retained; if the qualifiers differed (i.e., ‘=’ and ‘>’), the value that enabled unambiguous classification into either the toxic or non-toxic category was retained. For example: if two values were reported as LD_50_ > 90 and LD_50_ = 100 µg/bee, the latter (LD_50_ = 100) was retained; if two values were reported as LD_50_ = 200 and LD_50_ = 100 µg/bee, the latter (LD_50_ = 100) was retained.
- Data were excluded in cases where duplicate entries reported differing LD_50_ values and the ratio between the highest and lowest values exceeded 5.
- Data with values that could not be assigned to definitive toxicity were removed (e.g., cases where LD_50_ > 50 µg/bee were considered ambiguous).

**Figure 1:**
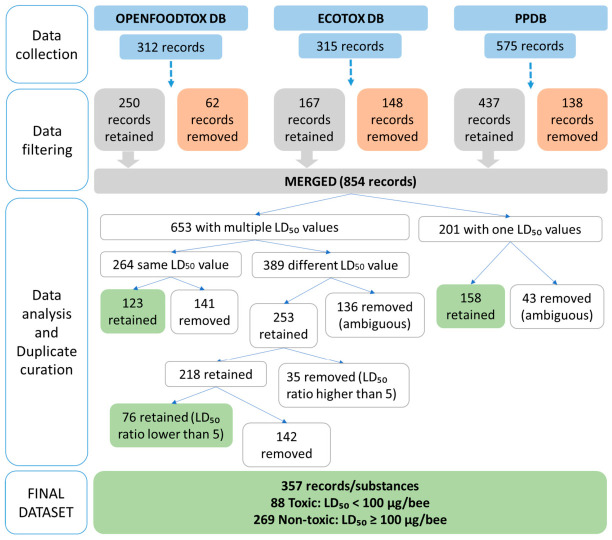
Schematic flowchart of the data curation pipeline. The workflow outlines the four sequential phases: initial data collection across the three source databases (OpenFoodTox, ECOTOX, and PPDB), data filtering based on specific criteria, duplicate curation, and the final distribution of the 357 unique compounds classified into toxic and non-toxic categories. Green boxes represent retained records/compounds, while orange boxes indicate excluded entries from the initial sources.

Figure 1 shows the workflow followed for data collection and data analysis in detail. The final dataset obtained by merging the data from the different sources comprised 854 records, after excluding data that did not meet the filtering criteria (Table 1, Table 2 and Table 3). Out of these 854 records:

- A total of 201 corresponded to unique substances with single LD50 values; 158 records were retained and 43 were removed for ambiguity:
- A total of 653 records were duplicates representing substances associated with multiple LD_50_ values. Out of the 653:
  ○ In total, 264 had identical LD50 values, from which 123 were retained and the remaining 141 were removed;
  ○ In total, 136 ambiguous records were discarded;
  ○ In total, 253 were evaluated based on the factor-of-5 rule. Out of this selection 218 records had an LD50 ratio lower than 5, resulting in 76 retained records and 142 were removed; whereas 35 records (corresponding to 8 substances) had a ratio higher than 5 and were removed.

### 2.3. Software and Modeling Methodology

#### 2.3.1. SARpy2.0

∆QSARpy (https://github.com/thedataconspiracy/DeltaQSAR, accessed on 7 January 2026) is an open-access computational tool designed for the automated identification of SAs [39]. It contains two modules, QSARpy to develop quantitative models using SAs, and SARpy2.0 to develop classification models using SAs. In this work, SARpy2.0 has been used to extract the SAs. When molecular datasets are provided in SMILES (Simplified Molecular Input Line Entry System) format, SARpy2.0 generates structural fragments and derives rules directly from the input data, encoding them in SMARTS (SMILES Arbitrary Target Specification) notation without reliance on pre-existing knowledge bases. The implemented algorithm uses the SMILES from the training set and generates substructures by testing every potential bond breaking. The generation of the substructures is the first step. The search is done by removing one atom from the SMILES structure, considering the resulting fragment. This is repeated for all atoms in a terminal position. From this initial, large fragment, the process is repeated sequentially in a similar way, removing the other atoms. In this way, a large number of fragments, even thousands, are generated. The second step is the evaluation of the individual substructures. This is done based on their F-score, which compares the predictive capacity of the fragments with the known activity (experimental class) of the compounds in the training set where the fragment is present. This evaluation is summarized via a confusion matrix. The final step is the extraction of the rules to get the final set of SAs. The SAs are selected sequentially, starting from the fragment with higher reliability. Considering this fragment, the software removes the substances associated with that fragment, obtaining a new, smaller dataset. Then, the algorithm progressively extracts less stringent fragments from the remaining dataset and calculates statistics on the smaller dataset. This process is applied sequentially. Therefore, the exact sequence is determined mathematically by the statistical prevalence of the fragments during this iterative extraction process. This procedure is different from other tools to identify fragments associated with the effect, because typically the process is done in a parallel way, and not in a sequential way, as in the case of SARpy. For this reason, these SAs should be evaluated and used jointly. The statistical significance of each extracted SA is quantified through a Positive Predictive Value (PPV), which serves as a statistical measure of its discriminatory power. Consequently, the output of SARpy2.0 consists of a set of statistically relevant SAs, each annotated with an activity label (e.g., active or inactive) and its corresponding PPV.

The training set has been used to extract the SAs for both classes (toxic and non-toxic) using the following settings:

- Fragmentation depth: 4
- MAX ERROR (defines the max error at which the selection of SA stops): 0.20
- BETA (precision/recall trade-off): 0.05
- SA without explicit hydrogen

All other settings were kept at their default values.

#### 2.3.2. istMolBase

istMolBase (https://chm.kode-solutions.net/pf/insilicotools-molbase/, accessed on 7 January 2026) is a Java-based in-house tool, designed for standardized molecular processing and structural analysis. It offers a suite of features, including SMILES/SDF parsing, salt neutralization, and standardized molecular normalization. For the purposes of this work, istMolBase was employed for its SMARTS matching capabilities, allowing for a rapid and systematic search of specific structural fragments within the dataset. SMARTS are the SMILES Arbitrary Target Specifications, which represent the substructure present in the molecule, using a format derived from the SMILES. This was necessary to simply identify the substances containing a given SA, standardize the SMARTS and make them ready for implementation in the VEGAHUB.

#### 2.3.3. Statistical Parameters

Model evaluation was performed using several statistical parameters. Overall predictive performance was assessed based on balanced accuracy (BA), which accounts for class imbalance by averaging sensitivity and specificity. Class-specific predictive ability was evaluated through sensitivity and specificity, which quantify the model’s performance for individual classes. The Matthews Correlation Coefficient (MCC) provides a global assessment of the model’s reliability by balancing all outcomes of the confusion matrix. In scenarios where the cost of a false negative (failing to identify a toxic compound) significantly outweighs that of a false positive, the F2 score was used. F2 score prioritizes the identification of toxic compounds by weighting sensitivity more heavily than precision.

The statistics were calculated with the following equations:

(1) $$\mathrm{BA}=\frac{\mathrm{sensitivity}+\mathrm{specificity}}{2}$$

(2) $$\mathrm{MCC}=\frac{\mathrm{TP}\times \mathrm{TN}-\mathrm{FP}\times \mathrm{FN}}{\sqrt{\left(\mathrm{TP}+\mathrm{FP}\right)(\mathrm{TP}+\mathrm{FN})(\mathrm{TN}+\mathrm{FP})(\mathrm{TN}+\mathrm{FN})}}$$

(3) $$\mathrm{Sensitivity}=\frac{\mathrm{TP}}{\mathrm{TP}+\mathrm{FN}}$$

(4) $$\mathrm{Specificity}=\frac{\mathrm{TN}}{\mathrm{TN}+\mathrm{FP}}$$

(5) $$\mathrm{Precision}\;(\mathrm{PPV})=\frac{\mathrm{TP}}{\mathrm{TP}+\mathrm{FP}}$$

(6) $$F_{2}\;\mathrm{Score}=\frac{\left(1+2^{2}\right)\times \mathrm{Precision}\times \mathrm{Sensitivity}}{{(2}^{2}\times \mathrm{Precision})+\mathrm{Sensitivity}}$$

TP means true positives (the toxic compounds predicted as toxic). FN means false negatives (the toxic compounds predicted as non-toxic). Similarly, TN indicates the true negatives (the non-toxic compounds predicted as non-toxic) and FP represents the false positives (the non-toxic compounds predicted as toxic).

## 3. Results

The initial phase of this study involved data collection from different sources and a cleaning and curation process to ensure the reliability of the predictive models.

**Figure 2:**
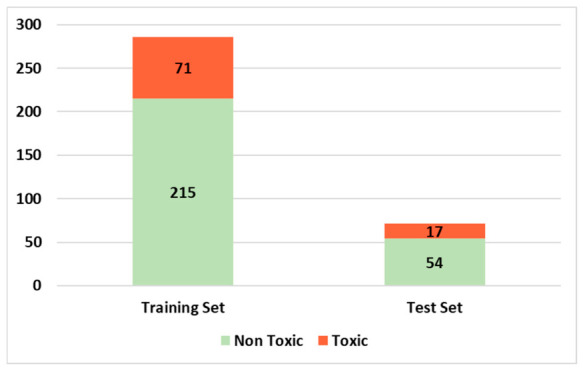
Bar chart showing the composition and distribution of the dataset used for model development and evaluation. The chart illustrates the total number of substances and the specific balance between toxic (red) and non-toxic (green) compounds across both the training set (left column) and the test set (right column).

**Figure 3:**
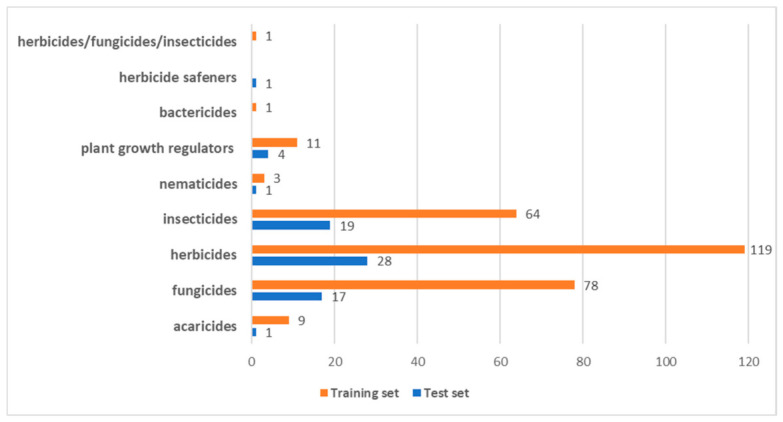
Graphical representation of the dataset splitting, showing the total number of pesticides assigned to the training set (orange) and the test set (blue), categorized according to their pesticide groups.

Data cleaning and curation led to a dataset of 357 records, 269 non-toxic compounds and 88 toxic compounds. A stratified split was used to divide the dataset and generate the training set (80% of the substances), used to train and develop the model, and the test set (20%), used to analyze the model’s performance on previously unseen data (Table S1). Figure 2 shows the splitting of the original dataset and Figure 3 shows the number of pesticides divided into their classes. In Table S1, for each compound, the pesticide group is reported.

### 3.1. Structural Alerts Extraction

The training set has been used to extract the SAs using SARpy2.0 with the settings mentioned in the Section 2. The corresponding sets of extracted rules are presented in Table 4.

### 3.2. Structural Alert Identification and Toxicity Prediction

The SAs reported in Table 4 have been used to create a classification model. Both the training and test sets were screened for the presence of specific SAs using the istMolBase. This analysis produced a matching matrix for each dataset, which lists whether a specific SA is present or absent within each compound (Table S2). To derive a final classification, a hierarchical ranking approach was applied: the 34 SAs were evaluated based on the numerical sequence assigned by SARpy2.0, and the first matching alert in the sequence determined the assigned class for the compound.

Table 5 reports the results. We notice that some substances have not been predicted (Table S3) because the software was not able to find any fragment to be used to classify the substance.

The results of the model are evaluated by comparing the training and the test set. In this study, we used balanced accuracy instead of accuracy, since accuracy only provides a measure of correct predictions and can be misleading in small and unbalanced datasets, as it does not differentiate between errors for toxic and non-toxic compounds [45]. The BA reached 0.92 in the training set and maintained a high value in the test (0.80). Looking at the specificity and sensitivity values, the model shows good performance in predicting the non-toxic class, obtaining a specificity of 0.95 in the training set and 0.82 in the test set. Similarly, for the sensitivity, results are good, although the statistics are slightly lower: the model reached a good performance for the training (0.89) and the test (0.79). This means that 80% of the hazardous compounds are correctly flagged as toxic, and this is fundamental from a regulatory and safety perspective.

The MCC provides a comprehensive measure that accounts for true and false positives and negatives, which is particularly useful in small or unbalanced datasets. The MCC dropped from 0.82 in training to 0.56 in the test set. While this decrease suggests that some SAs are highly specific to the training set, an MCC of 0.56 still indicates a good predictive model. This MCC drop highlights that out of the applicability domain (AD), defined by the SAs used, the models can have a lower performance.

Finally, since in a regulatory point of view the identification of the toxic class is important, the F2-score was used to prioritize the FN rate and the identification of the toxic class. The model achieved an F2-score of 0.74 in the test set. This confirms the model’s reliability in a “conservative” approach, prioritizing the identification of hazardous compounds. Despite a lower Precision (0.61), caused by 7 FP, the low number of FN ensures that the model remains a useful tool for preliminary toxicological assessments.

## 4. Discussion

### 4.1. Performance of the Models

In this paper, a classification model to predict oral acute toxicity in *A. mellifera* was developed and presented. The model was obtained using a set of SAs, extracted from SARpy2.0 and analyzed with istMolBase. To ensure high predictive reliability, the model was trained on a robust dataset obtained from three sources, including high-quality verified data from the OpenFoodToxDB. To maximize data quality, all records underwent a rigorous curation and filtering workflow, removing noise and inconsistencies to yield a highly refined dataset. Furthermore, the toxicity thresholds applied in this study strictly adhere to official European regulatory standards, ensuring that the model’s classifications are directly relevant to current ecotoxicological frameworks. The model demonstrated satisfactory predictive performance, despite the class imbalance, with a balanced accuracy of 80%. The overall statistics of the model showed a good ability to discriminate between classes, reaching a sensitivity and specificity of ~80%. The model’s ability to correctly predict the compounds is strictly dependent on the AD due to the SA approach used. This dependency is reflected in the MCC that, while remaining moderately good, dropped to 0.56 in the test set. Despite this, the F2-score of 0.74 in the test set highlights a good prioritization of the toxic class, effectively minimizing FN. The model achieved a test set precision of 0.61, meaning that around 39% of the compounds predicted as toxic are false positives. In a regulatory screening context, this trade-off is justified by the precautionary principle. Both Regulation (EC) No 1107/2009 and the EFSA Guidance [17] focus on avoiding risk underestimation. Minimizing false negatives is more important than avoiding false positives. Underestimating the toxicity of a pesticide poses a real threat to honey bee populations. A precision of 0.61 shows that the SAs are sensitive enough to catch potential hazards; therefore, a QSAR model that accepts more false positives to avoid missing toxic compounds is consistent with EU regulatory expectations for bee protection. Consequently, the model proves to be a conservative and competitive primary filter for preliminary toxicological assessments.

To enable a meaningful comparison with other models available in the literature, it is important to note that several in silico models have been reported in the literature for the prediction of acute contact toxicity of compounds in honey bees (*A. mellifera*) [30,31,33,34,46,47,48]. In contrast, the number of models addressing acute oral toxicity remains limited at the time of this study. A notable example is BeeToxAI [49], an artificial intelligence–based web application developed to evaluate the acute toxicity of chemicals in honey bees. For model construction, the authors compiled both oral and contact acute toxicity data and applied machine learning algorithms (Random Forest and Support Vector Machines) in combination with three molecular fingerprint types (MACCS, FCFP, and ECFP). Among these, the MACCS + RF model yielded the highest predictive performance on the external validation set (Accuracy = 0.88; Sensitivity = 0.86; Specificity = 0.90; MCC = 0.76). It should be noted, however, that unlike our approach, BeeToxAI employed a different classification threshold (11 µg/bee) following the EPA guidance [18]. A comparison of the model described in this paper and BeeToxAI is reported in Table 6. BeeToxAI gives better statistical values. However, its applicability is probably more limited compared with the model described here, which has more than double the number of substances. Thus, the acquisition of additional, high-quality oral toxicity data represents a key step toward enhancing the robustness and validity of the model. Our model uses the threshold values applied within regulatory frameworks [17,20], which is an advantage for its direct application. Our model is relatively simple, since it only refers to the SMILES and fragments generated by this format, while BeeToxAI applies random forest algorithms.

### 4.2. Interpretation of the Structural Alerts

The SARpy2.0 model identifies fragments present in the substances in the training set which are associated with both toxicity and lack of toxicity. Depending on the setting of the software, SAs only found in one class (PPV = 1), or SAs which can be used to separate both classes (PPV < 1) can be obtained. The settings depend on the users’ priorities (precision vs. coverage vs. interpretability) and dataset. Considering the fragments in Table 4, several SAs are associated with toxicity, and a higher number are associated with lack of toxicity. The majority of SAs are associated with lack of toxicity since the dataset is unbalanced towards the non-toxic class. The SAs related to toxicity refer to the presence of fragments which are known to be related to adverse effects. Some of the SAs associated with toxicity are only present in toxic compounds. For example, the SA1 identifies the organophosphate compounds, and SA30 identifies thiophosphate compounds. Both classes are known for their acetylcholinesterase inhibition power. SA1 fragment is present in seven pesticides in the training set, all of which are toxic: Fenamiphos, Cadusafos, Ethoprophos, Oxydemeton-methyl, Chlorfenvinphos, Dicrotophos, and Monocrotophos. SA30 is present in six compounds of the training set; five out of six are toxic: Diazinon, Dimethoate, Phosmet, Chlorpyrifos-methyl, and Malathion. SA2 is found in six compounds that are pyrethroids: Tefluthrin, Bifenthrin, Fluvalinate, Acrinathrin, Cyfluthrin, and Momfluorothrin. SA3 can represent neonicotinoids and is found in five molecules with high toxicity: Imidacloprid, Thiamethoxam, Imidacloprid olefin, Dinotefuran, and Clothianidin. SA8 can be found in pyrazole compounds; it is found in five toxic molecules in the training: Tetraniliprole, Fipronil, Tebufenpyrad, Fipronil sulfone, and Cyclaniliprole. In the test set, this SA wrongly predicts two non-toxic compounds: 5-(Trifluoromethyl)-1H-pyrazole-3-carboxylic acid and Chlorantraniliprole.

There are a few tens of SAs associated with lack of toxicity. This fact is linked to the higher number of non-toxic substances present in our dataset. While in the case of SAs associated with adverse effects it may be easier to recognize a structure known to be toxic through a known mechanism, in the case of SAs associated with lack of effect the reasons are more complex. It may be that certain moieties block the effect, or in other cases kinetic processes intervene. For instance, there are polar groups which may reduce the lipid absorption. Seven of the SAs are amides, mainly aromatic ones. Heterocyclic residues appear quite frequently. Some SAs contain an aromatic ring with the trifluoromethyl residue.

SARpy considers the position of the substituents in the ring, and thus if the trifluoromethyl, for instance, is in a different position relative to the nitrogen, the fragment does not match with the SA.

SARpy2.0 identifies moieties in a sequential and not parallel way. It means that initially the software searches for a large fragment that has a large percentage of association with effect or lack of effect. The substances containing this fragment are eliminated because the reason for their effect (or lack of effect) has been identified, and the search starts again, sequentially. Progressively, the last substances will be with mixed activity. Thus, the software identifies a sequence of fragments, and all of them are used to label substances. As a consequence, all SAs should be used and evaluated jointly, and not individually. For instance, certain SAs that appear quite generic should be interpreted as a way to predict the substances that have not been explained by the presence of the previous SA. Thus, the alerts are context-specific, and not general ones. Another aspect is that these alerts apply to pesticides, and in this context the model is expected to give better results.

The thresholds to accept fragments as useful for modeling purposes are defined by the modeler. In our case, we obtained (after many attempts) the best models for our purpose using the threshold mentioned in the Section 2. The SAs obtained in some cases have a PPV below 1 but above 0.8 (less stringent requirements); thus, fragments with a prevalence up to 80% of toxic or non-toxic substances are accepted. The use of these additional SAs allows for processing a larger number of substances. As we explained, SARpy2.0 extracts progressively rules for effect. The model itself is a collection of SAs which should be used jointly. The SAs which have been extracted as the last ones (PPV < 1) are present in both toxic and non-toxic substances. Theoretically, it would not be correct to use a single SA individually as a model, since they must be used jointly. Practically, some SAs may generate false positives or false negatives, and thus it would be wrong to understand that the presence of a certain fragment is associated with a 100% probability of effect.

In this study, a hierarchical approach was employed to predict compound toxicity, following the ranking of SAs established by SARpy2.0. The SAs extracted during this analysis were integrated into istMolBase to perform the structural matching. The resulting confusion matrix was used for the final predictions. Accordingly, the prediction for a given compound is determined by the first matching SA encountered in the prioritized list. In case a substance contains conflicting SAs, the hierarchical approach resolves this by selecting the SA with the highest reliability score.

However, alternative strategies may be implemented using all or some SAs, depending on the predictive stringency:

- Consensus Approach: All SAs present within a single molecule are identified, and the final classification is derived by the most frequent category.
- Conservative Approach: A compound is predicted as toxic if it contains at least one SA associated with toxicity.

This modularity ensures that, depending on the specific regulatory or research objectives, the set of SAs can be rearranged or the decision logic modified to optimize performance for acute oral toxicity assessments. It is up to the user, depending on the specific purpose of the study, to apply a conservative strategy or prefer the accurate value. Still, it may be that a substance does not contain a fragment from the lists of SAs. In this case, the model cannot make a prediction.

### 4.3. The Evaluation of the Models According to the OECD Principles

We presented a new classifier for the evaluation of toxicity of pesticides towards honey bees. The first principle requires that the endpoint is clearly defined. Indeed, the classifiers are specific for oral acute toxicity (LD_50_) towards *A. mellifera*. The threshold value is defined as 100 µg/bee. The second principle refers to the presence of an unambiguous algorithm. The software has been described, and the model, which utilizes SARpy2.0, is based on the identification of a clearly defined list of SMARTS to be used for labeling pesticides as toxic or not. Any user will obtain exactly the same results. The third principle addresses the AD. The AD of the proposed model requires a fundamentally different conceptual approach compared to the traditional one, where the AD is defined within a continuous, multidimensional chemical space. In such frameworks, a test compound is flagged as out-of-domain based on its distance (e.g., Euclidean or Mahalanobis distance) from the training set cluster or based on molecular descriptors. Conversely, our model relies on a rule-based AD strictly defined by the presence or absence of the extracted SA. A molecule is considered inside the AD (predicted) only if it contains at least one of the SAs generated during the training phase. If a compound does not contain any SAs, it is classified as “not predicted” (out of domain). Considering the substances out of the AD found by the model, several of them have rings with heteroatoms. As we explained, SARpy is quite precise in the topology of the fragment; thus, if the ring is not exactly as in the SA, the software will not assign a label for effect. Similarly, several substances out of the AD contain fluorine atoms. In Table 4, there are two SAs containing a trifluoromethyl residue linked to a pyridine or aniline ring. If the fluorine atoms are in a different moiety, or even in the same ring but in a different position, the software will not assign the label which is specific for the structure containing that SA. In other terms, SARpy requires an exact match, and related or more generic fragments are not considered reliable and useful for classification.

The model is specific for pesticides, and more specifically, it is requested that certain molecular features, defined through SMARTS, are present in the molecule. If the pesticide does not contain any of them, the software does not make a prediction, and indeed some substances have not been classified (see Table 5). Applying the wording of the in silico models, these substances are out of the AD of the model. We provided statistical parameters related to both the robustness and the predictivity of the model, as requested by the fourth principle. Finally, we discussed the mechanistic reasoning, with reference to the fifth principle.

### 4.4. Advantages and Disadvantages of the Proposed Models

The proposed model has good performance, as we discussed above. The model is quite simple, since they only require the SMILES of the structure. This is an advantage compared to other more complex models, which require calculation of molecular descriptions, sometimes three-dimensional ones. The algorithm, once the model is built up, is very simple and only consists of a sequence of SMARTS, codifying the molecular fragments. Another advantage is that these fragments can be easily read and understood in their chemical composition. Overall, the proposed model can be useful both to screen new pesticides and to plan safer alternatives. The model is fast, does not require the synthesis of the substance, and can predict batches of substances. Industry may apply this before the real synthesis of new substances. Furthermore, the model listed molecular moieties associated with toxicity or lack of toxicity. These moieties can be useful in the design of new pesticides.

The disadvantages are related to the reduced amount of data, which covers several categories of pesticides, but not all of them. Future pesticides with completely different structures may not be predicted, and the model could not offer reliable results. We notice that these limitations apply not only to our model, but also to all in silico models aiming to address the same endpoint. Regarding the interpretation of the mechanism, at the present time the fragments should be used to investigate possible mechanisms, but further studies are necessary to demonstrate the real mechanism.

## 5. Conclusions

The risk posed to pollinators is of great concern, and the application of NAMs for assessing chemical hazards offers a cost- and time-efficient alternative to traditional laboratory testing. In this study, we developed a classification model to predict the toxicity of pesticides towards *A. mellifera*, categorizing them as toxic or non-toxic. The model was constructed using SARpy2.0 and trained on a dataset of acute oral LD_50_ values for *A. mellifera*, compiled from both open-source and commercial databases, including OFT, ECOTOX and PPDB. These findings demonstrate the potential of computational approaches to support risk assessment and contribute to the protection of essential pollinator species. The developed structural alert-based model provides a reliable and balanced approach for the toxicological screening of pesticides. While the model achieves a Balanced Accuracy, Sensitivity and Specificity of ~80%, its performance is constrained by the Applicability Domain. This chemical space dependency is evidenced by a low Precision of 0.61 on the test set (resulting in around 39% false positives) and a corresponding drop in the MCC to 0.56. Consequently, this model should not be used as a standalone decision tool; to increase confidence, other lines of evidence should be added, such as the evaluation of toxicity of related substances. Anyhow, even in its present stage, the model is highly suited as a preliminary screening tool. The high F2-score confirms the model’s success in prioritizing the identification of hazardous substances. By minimizing FN, this model serves as a conservative tool for preliminary risk assessment and the prioritization of compounds for further experimental testing.

The proposed model, beyond sound statistical performance, identified structural fragments associated with the adverse effect, allowing reasoning about the role of certain structural moieties. These moieties are also used by the model to define the AD of the model. Overall, this approach could optimize both predictive reliability and coverage in toxicological classification.

Although the proposed SAR model demonstrates high transparency and good predictive performance for regulatory screening, further refinements could be explored in future studies. Future investigations should focus on expanding the SA library by continuously integrating newly published data, thereby broadening the model’s applicability domain. An improvement can be made by combining these rule-based SAs with other machine learning approaches, using additional toxicological information on bees, such as information on metabolic activation, accounting for biotransformation pathways that might generate toxic metabolites from benign parent compounds. Lastly, in vitro assays could be designed to experimentally validate the biological mechanisms hypothesized by the extracted SAs.

While moving from a binary classification threshold to a continuous regression model represents a desirable methodological evolution, such a transition is currently restricted by data-availability constraints. Indeed, ecotoxicological repositories for *Apis mellifera* frequently report endpoints as open-ended ranges (LD_50_ < 30) rather than exact, continuous numerical values.

The model will be implemented on the VEGAHUB website (www.vegahub.eu) for free use; this will allow for the exploration of similar related substances to be used as an additional line of evidence in the overall assessment.

## Figures and Tables

**Table 1:** Filters applied to the OFT database.

Test type	Acute toxicity
Species	Honey bees
Route	Oral feed; oral unspecified
Dose unit	µg/piece
Endpoint	LD50
Exposure	≤4 days; empty cells were maintained
Subtype	Single chemical entity
Qualifier	As such
Com_type	Organic

**Table 2:** Filters applied to the ECOTOX database.

Species Scientific Name	*Apis mellifera*
Organism life stage	adult; empty cells were maintained
Exposure Type	Food; oral via capsule; diet
Exposure Duration Unit (Days)	≤4 days; empty cells were maintained
Conc 1 Type (Author)	Active ingredient
Dose Units (ug/org converted in µg/bee)	ug/org * mg/org AI ug/org * mg/bee AI mg/org * ng/org ae ug/org * AI ng/org * pg/org ug/bee *
Test Type	Acute

* AI = Active ingredient; ae = acid equivalent; ug = µg.

**Table 3:** Number of records collected and filtered.

Database	Initial Records	Records Filtered
OFT	312	250
ECOTOX	315	167
PPDB	575	437

**Table 4:** Rules extracted with SARpy2.0.

SA Number	PPV	CLASS	SMARTS
SA1	1.00	TOXIC	COP=O
SA2	1.00	TOXIC	CC(CC(=O)OCc1ccccc1)C
SA3	1.00	TOXIC	[O–[N+](=O)N
SA4	1.00	NON TOXIC	CC(=O)Nc1ccccc1
SA5	1.00	NON TOXIC	OC=CC=O
SA6	1.00	NON TOXIC	COCCOc1ccccc1Cl
SA7	1.00	NON TOXIC	Cc1cnn(c1)C
SA8	1.00	TOXIC	Cc1ccnn1
SA9	1.00	NON TOXIC	Nc1cccc(c1)O
SA10	1.00	NON TOXIC	CSC=O
SA11	1.00	NON TOXIC	FC(c1cccnc1)(F)F
SA12	1.00	NON TOXIC	NC=C(C)C
SA13	1.00	NON TOXIC	O=CNc1cccc(c1)Cl
SA14	1.00	NON TOXIC	ClCCl
SA15	1.00	NON TOXIC	O=C(c1ccccc1)NC(C)(C)C
SA16	1.00	NON TOXIC	Nc1cccc(c1)C(F)(F)F
SA17	1.00	NON TOXIC	CC(=O)NCc1ccccc1
SA18	1.00	NON TOXIC	OC(=O)Cc1ccccc1
SA19	1.00	TOXIC	CCc1cccc(c1)C
SA20	1.00	NON TOXIC	O=Cc1ccccn1
SA21	1.00	NON TOXIC	CC(=O)NN
SA22	1.00	TOXIC	CSC=N
SA23	1.00	NON TOXIC	Nc1ccccc1Cl
SA24	0.88	TOXIC	CCCc1ccccc1
SA25	1.00	NON TOXIC	Cc1ccccc1O
SA26	1.00	NON TOXIC	COCN
SA27	1.00	NON TOXIC	O=CNc1ccccc1
SA28	0.88	NON TOXIC	COc1ccncn1
SA29	0.83	NON TOXIC	CCCCCO
SA30	0.83	TOXIC	COP=S
SA31	0.86	NON TOXIC	c1ccncn1
SA32	0.83	NON TOXIC	CCNC=O
SA33	0.80	NON TOXIC	CCOC(=O)CO
SA34	0.80	TOXIC	Cc1ccc(cc1)O

**Table 5:** Statistical results of the training and test sets.

Training Set						Test Set				
	Pred	Toxic	Non Toxic	Not Predicted			Pred	Toxic	Non Toxic	Not Predicted
Class						Class				
Toxic		47	6	26		Toxic		11	3	3
Non Toxic		9	180	18		Non Toxic		7	32	15
Balanced Accuracy				0.92		Balanced Accuracy				0.80
MCC				0.82		MCC				0.56
Specificity				0.95		Specificity				0.82
Sensitivity				0.89		Sensitivity				0.79
Precision				0.84		Precision				0.61
F2-Score				0.88		F2-Score				0.74

**Table 6:** Comparison with BeeToxAI model.

	BeeToxAI	This Paper’s Model
Data sources	Literature OpenFoodTox ECOTOX OCHEM (Online Chemical Modeling Environment)	OpenFoodTox ECOTOX, PPDB
Dataset	Oral exposure: 169 (71 toxic and 98 non-toxic)	Oral exposure: 357 (88 toxic and 269 non-toxic)
Classification thresholds	11 μg/bee	100 μg/bee
Modeling approach	SVM, RF combined with molecular fingerprint (MACCS, FCFP and ECFP)	SARPY2.0 + istMolBase
Statistics	Statistics on the external validation, (MACCS + RF) Accuracy = 0.88 Sensitivity = 0.86 Specificity = 0.90 Cohen’s k = 0.76	Statistics on the test set: BA = 0.80 MCC = 0.56 Specificity = 0.82 Sensitivity = 0.79 Precision = 0.61 F2-Score = 0.74

## Data Availability

The original contributions presented in this study are included in the article/Supplementary Materials. Further inquiries can be directed to the corresponding author.

## Supplementary Materials

The following supporting information can be downloaded at: https://www.mdpi.com/article/10.3390/jox16040130/s1.
